# The psychology of “cure” - unique challenges to consent processes in HIV cure research in South Africa

**DOI:** 10.1186/s12910-019-0348-z

**Published:** 2019-01-24

**Authors:** Keymanthri Moodley, Ciara Staunton, Theresa Rossouw, Malcolm de Roubaix, Zoe Duby, Donald Skinner

**Affiliations:** 1 Centre for Medical Ethics and Law, Department of Medicine, Faculty of Health Sciences, Stellenbosch University, Stellenbosch, South Africa; 2 Institute for Cellular and Molecular Medicine, Department of Immunology, University of Pretoria, Pretoria, South Africa; 3 HIV AIDS STDs and TB, Human Sciences Research Council, Cape Town, South Africa; 4 Dept. of Global Health, Faculty of Medicine and Health Sciences, Stellenbosch University, Stellenbosch, South Africa

**Keywords:** HIV, Cure, Consent, South Africa, Stakeholders

## Abstract

**Background:**

Consent processes for clinical trials involving HIV prevention research have generated considerable debate globally over the past three decades. HIV cure/eradication research is scientifically more complex and consequently, consent processes for clinical trials in this field are likely to pose a significant challenge. Given that research efforts are now moving toward HIV eradication, stakeholder engagement to inform appropriate ethics oversight of such research is timely. This study sought to establish the perspectives of a wide range of stakeholders in HIV treatment and research to inform consent processes for cure research.

**Methods:**

In total, 68 South African stakeholders participated in two qualitative research modalities. In-depth interviews (IDIs) were conducted with a purposive sample of 42 individuals - audiotaped with consent. Twenty-six stakeholders participated in three focus group discussions (FGDs). Thematic analysis of transcribed IDIs and FGDs was conducted.

**Results:**

The majority of respondents indicated that there could be unique challenges in HIV cure research requiring special attention. In particular, given the complexity of cure science, translation of concepts into lay language would be critical for potential participants to adequately appreciate risks and benefits in early phase research with experimental interventions. Furthermore, to aid understanding of risks and benefits against a background of desperation for a cure, specially trained facilitators would be required to assist with a psychological assessment prior to consent to avoid curative misconceptions. Long-term participant engagement to assess durability of a cure would mean that the consent process would be prolonged, necessitating annual re-consent. Building trust to maintain such long-term relationships would be critical to retain study participants.

**Conclusion:**

Unique consent requirements for cure research in South Africa would include significant efforts to maximise understanding of trial procedures, risks and the need for long-term follow-up. However, the psychological dimension of cure must not be underestimated. Beyond an understanding of cure science, the emotional impact of HIV cure advances the discourse from cure to healing. Consequently, the consent process for cure research would need to be enhanced to include psychological support and counselling. This has several important implications for research ethics review requirements for consent in HIV cure research.

## Background

The science of HIV cure research is complex. Potential cure strategies include the use of highly active antiretroviral therapy (HAART) during acute infection, latency-reversing compounds, gene editing, the administration of broadly neutralizing antibodies, therapeutic vaccines, and/or various combinations of the above. Some of these strategies will include analytical treatment interruption [1]. At least a hundred early phase trials have been completed in the United States relating to various aspects of HIV cure research [2]. Approximately a hundred additional trials are in progress [3]. In South Africa, cure research started in children in 2017 using the strategy of very early treatment (personal communication). Data from this research has not been published as yet.

Historically, research on most other diseases has been framed as “treatment” research, and not cure [4] In diseases like Syphilis, treatment evolved into cure [5, 6]^.^ Experimentation occurred on a trial and error basis and not in large scale randomised controlled “cure” trials as is currently the plan for HIV eradication.

The uniqueness of AIDS ‘cure’ research does not lie in the meticulous ethical underpinning of the research as described elsewhere, such as the quest for authentic informed consent, problematic as it may be. To an extent it lies in the nature, epidemiology and politicisation of the disease and consequent trial design, but perhaps even more in the socioeconomic situatedness, extreme vulnerability and disempowerment of likely participants in developing countries and the value laden societal judgements and stigmatisation of those afflicted by HIV and AIDS.

This vulnerability has already been exploited by several offers of cure in the past. Such offers either pre-dated the availability of antiretrovirals or coincided with the experience of severe adverse effects by patients on antiretroviral treatment. It is unsurprising that the concept of “HIV cure” in South Africa is contentious given the wide range of illegitimate and fraudulent cures for HIV that have been advanced over the past three decades. Such “cures” include herbal, traditional and chemical compounds offered to vulnerable patients by charlatans, politicians, healthcare workers and scientists, both local and foreign [7–12].This complicates, confounds and undermines the legitimacy of the current scientific agenda of HIV eradication.

A wide range of ethical considerations have recently emerged in the limited literature on HIV cure research [13–18].In particular, concerns have been raised about the consent process - how will trial information be communicated to participants and patients, what will motivate participation in potentially high risk research with little prospect of direct benefit and how will understanding of trial procedures, risks and benefits be assured? [2, 19, 20]. Analytical treatment interruption evokes concerns about viral rebound and increases the risk profile associated with HIV eradication studies [1]. In addition, the language used in communicating “cure” as a concept is challenging in many respects [21]. Differentiating between a “functional” cure and a “sterilizing” cure is of paramount importance in the consent process [22]. Some of these ethical concerns have been raised in the context of Hepatitis B cure research in resource rich settings. While HIV is a multisystem disease, hepatitis affects the liver primarily, so scientifically, the research challenges are different. Ethical concerns may be similar in some respects but differ in others [23].

The ethical concerns raised by HIV cure research are likely to be exacerbated in resource poor settings, where HIV cure research has not started in earnest, and where consent processes on less complex research are already challenging [24–27] . While significant efforts have been invested in developing consent processes and tools for HIV prevention and treatment research in South Africa over the past three decades [28–31], work on HIV cure consent processes remains in its infancy [26, 32]. Given the high burden of disease due to HIV in South Africa, a “cure” is imperative as a medical breakthrough. However, the history of HIV research in this country has taught us that engaging with a broad range of stakeholders via formative research ahead of the science is important to establish perceptions around how “cure” is understood, how understanding of complex science can be facilitated and how risk assessment can be facilitated so that informed consent is authentic. This is particularly important against the historical context of illegitimate cures in South Africa [7–12].

As part of a broader study on the intended and unintended consequences of HIV cure research, this paper specifically explores the perspectives of multiple, diverse stakeholder groups on the anticipated challenges in HIV cure research in South Africa to guide the development of consent processes and protocol review by research ethics committees (RECs).

## Methods

In order to explore some of the pertinent intended and unintended consequences of HIV cure research in South Africa, we conducted individual in-depth interviews (IDIs) and focus group discussions (FGDs) with a broad range of stakeholders with experience of working or living with HIV in South Africa.

### Sample

Using purposive or strategic sampling, making a deliberate choice of respondents to ensure coverage of as full a range of characteristics of interest as possible [33, 34]; we selected respondents from a range of stakeholder categories to elicit diverse commentary on the potential ethical issues around HIV cure research. The respondents included scientists involved in different stages of cure research, from the laboratory to clinical trials, clinical professionals from academic, public and private health services, social scientists who work in the area of HIV, representatives from RECs, religious figures with a community profile, activists working in HIV, medical students and patients. This provided a wide range of potential opinions within the South African context. Many of the respondents have international profiles which we feel will enhance the value of the results to contexts outside of South Africa.

The total sample for this project included 83 stakeholders who were contacted telephonically or via email. Of these, 42 agreed to be interviewed (IDIs) and 15 declined participation or did not respond to a request for an interview (IDIs). All participants who were approached to participate in FGDs, agreed (*n* = 26). Three FGDs took place: two with people living with HIV (PLHIV) including caregivers of children living with HIV, and a third with medical students.

**Table:** 

Table of respondents	
Laboratory scientists	5
HIV trial researchers	5
Academics	8
Public health administrators	3
Private Health Funders	5
Social scientists	5
Ethics committee members	4
HIV activists	4
Religious leaders	2
Journalists	1
FGD1: Medical Students	6
FGD2: PLHIV	9
FGD3: PLHIV	11

Material from 14 of the IDIs with HIV experts was analysed and published previously [32]. This paper represents the views of 54 participants (28 other HIV stakeholder groups obtained via IDIs and 26 FGD participants) and reflects key themes relating to consent specifically that had not been addressed in the earlier paper.

### Fieldwork

The IDIs were conducted by seven researchers in four different cities in South Africa (Cape Town, Johannesburg, Pretoria and Durban) and two of the seven were involved in the FGDs. Interviews were conducted after informed consent was obtained and each lasted approximately 40–60 min. All IDIs were conducted in English and the FGDs were primarily in English. For interviews with PLHIV and caregivers, an interpreter was available for isiXhosa and Afrikaans speaking patients.

The interviewers had diverse professional backgrounds: bioethicist, lawyer, social scientist, genetic counsellor, clinician, psychologist and medical scientist. The methodological approach was contextualised as interpretative research, to achieve a deeper understanding of what the respondents were saying, and the values and paradigms that guided their thinking [35, 36]. All interviewers were trained on the objectives of the study prior to going into the field and shared a common understanding about the focus of the interviews. The discussion schedule used for all interviews included the following broad areas: HIV Cure Concepts, History of HIV Cure, Cure Research Ethics, Cure Research participation, Cure Early Implementation, and Internet Access. The emphasis given to each section would differ by participants and not all areas are reported in this paper, as this paper is only part of a broader study.

The interviews were framed using the following context: interviewers explored perspectives around ethical and social issues associated with hypothetical functional and sterilizing “cures”. In either case, a hypothetical “cure” intervention study to assess impact would require an extended period of follow-up and monitoring to ensure that the “cure” had worked and that all the body’s reservoirs were cleared of disease. The participants recruited in such hypothetical trials would include both those who were newly diagnosed with HIV and those who had been on HAART for an extended period with a controlled HIV viral load in the blood. The focus was on hypothetical adult patients recruited from treatment or testing clinics in geographically defined communities in South Africa. The research design assumed was that of a phase 3 randomised control trial to assess effectiveness of a new cure intervention. Interviewees understood that, given the early stage of cure research, many of the early trials are likely to be unsuccessful and it may take a while to develop an effective and usable “cure”. This scenario is analogous to what is happening with microbicide and HIV vaccine research. A plain language fact sheet was provided to all participants at the start of the interview, briefly summarising HIV cure concepts and research, focusing on the information provided above.

### Analysis

Recorded interviews were transcribed verbatim. Formal analysis began two thirds of the way into the data collection. Two of the interviewers (ZD and CS) took a lead in developing the codebook based on detailed preliminary readings of the initial interviews. They also consulted other members of the team including DS who was leading the formal analysis and who had read the interview transcripts. Emerging themes were discussed between the principal investigator (KM) and other members of the team (DS, CS). To maximise inter-coder reliability, codes were defined clearly and distinctively. Three competent coders coded all the transcripts and reconciled coding discrepancies. The coding was refined until an acceptable degree of inter-coder reliability was reached. Throughout this process, codes were reviewed to assess the necessity of adding additional codes. No additional codes were added, as all the data fitted into existing codes; however some definitions were altered as the coding progressed, to take the full extent of the data into account. Data analysis was facilitated by using the software program Nvivo. A contextualised thematic approach, in which the quotes associated with each theme are interpreted in terms of the context in which they are raised [33, 35, 36], was used to interpret the results. DS lead the analysis but met regularly with CS and KM to discuss results and check the interpretations made. A specific separate analysis was not done for each stakeholder group, as the focus was on finding common understandings across all the groups. Variation across the stakeholder groups was noted, especially where one group took a different stand on a particular issue. Where this impacted on the discussion, it was noted in the text.

### Ethics

This study was approved by the Health Research Ethics Committees at Stellenbosch University (N13/05/063), the University of Pretoria (29/2015) and the University of Cape Town (761/2014). In addition, provincial approval was obtained where required (Tygerberg Hospital). All participants signed an informed consent form stating that they understood the research and that they agreed both to be interviewed and to audiotaping. All personal identifying data were removed from the interviews.

## Results

Six main themes emerged from the data. The key discussion focused on the way in which HIV cure research differed from other clinical trials, and the risks that this may expose research participants to. The discussion is framed around the construct that researchers are obliged to protect participants from dangers that may not be realised or that are difficult to conceptualise.

The emergent themes are discussed in detail below:

### HIV exceptionalism and consent

In any clinical trial, provision of informed consent prior to enrolment is a pre-requisite to participation. The standard procedure is well outlined in Good Clinical Practice (ICH-GCP) guidelines for clinical trials and in various other research ethics guidelines. A central theme throughout the interviews was that these guidelines should direct HIV cure trial research and that some special additional considerations need to be applied to ensure complete understanding. There was also concern that HIV has, at times, been treated as “different”.

All clinical studies should be done exactly the same. (L = REC member)

I am not too sure why people see it [HIV] as (different); or what people foresee as an ethical difficulty. I mean as long as the agent follows the normal process of what clinical trials do, and the safety considerations of clinical trials. As long as it follows the normal processes; I am not quite sure what the ethical dilemmas even are. (G = public health administrator)

Having said that, the dominant narrative identified areas within the current requirements that do need particular attention in the case of HIV cure given the desperation and vulnerability of many HIV-infected participants in the South African context:

I think there needs to be a lot more dialogue than normally would happen. I mean because of all the attention around the illness. (A= private health funder)

Well, consent is basic …and I think that it is problematic - to really make sure that people are well informed … as people may sort of grab at the last straw to say “Well let’s try it” just out of being desperate and not really being well informed. (M = religious leader)

### Improving understanding through science translation

Respondents expressed concern over scientifically complex patient information leaflets and consent documents:

The lengthier and the more detailed the informed consent process is, the more likely the patients are to switch off. So it's easier for them to understand one page than it is to understand – I mean some of the pharmaceutical trial informed consent documents are like mini-theses; and you get tired just looking at them and they tend to be very repetitive, not easy to read and all sorts of clauses which are more designed to protect the researchers and the sponsor, than... are really of benefit to patients. (B = academic)

Likewise, many of the constructs used in the explanation of cure are difficult to understand. For example, remission has been suggested as a suitable alternative to the word “cure”. However, it can be difficult to communicate; metaphors such as talking about remission in cancer are not necessarily accurate and carry loaded meanings depending on the person’s experience and awareness. Very few people, even in research and clinical settings, fully understand the meaning of the words remission, functional cure or reservoirs, amongst others. These are all words that cure research uses extensively and will require accurate, yet simple, explanation.

I've got a PhD and I work in health research; I don't really know what remission means… and I'm English speaking; but I still think in our context we have to deal with many, many issues in clinical trials that people struggle to understand… but we’ve got to keep trying. So… who understands randomization; who understands prophylaxis? (aa = social scientist)

The level of explanation required did vary across the sample that we interviewed, with some respondents feeling that the standard GCP requirements were sufficient with a good assessment of knowledge. Others argued that a far more detailed profile and assessment of the readiness of potential participants was necessary.

It would have to be quite an extensive psychological evaluation on these potential candidates … where it’s fully explained to them… what the risks are and then somebody – a psychologist or somebody would have to determine ″Okay, this patient is fully aware of everything and he’s in a position – a stable position where he would happily make this decision, and he can cope with the consequences. (FGB2 = medical student)

The informed consent is sort of providing the support framework, so it is the same as when you do any of the psychological studies that you have built in; that there is recourse to support. And this – if it’s built in from the beginning that there are regular check-ups to assess mental state and understanding. (D = REC member)

A key point raised was that the processes by which people construct meaning based on what they are told are poorly understood. As with any other new or different construct people may encounter, they tend to reconstruct it in terms that they understand. This background knowledge used to construct meaning cannot always be known by the research team, so achieving informed consent may require an active process to explain the study, to engage and work with the potential participant until an adequate level of understanding has been achieved. The potential for confusion and misunderstanding of the implications of participation is immense. Participants may be getting different messages and information from other healthcare providers during the trial, as well as from non-medical sources such as friends, traditional and alternative healers, family members and the media.

We know that memory isn't recall, it's a reconstruction; so all of this is going to get reconstructed and changed and reconfigured. (aa = social scientist)

### Communicating risks and benefits accurately

A strong consistent recommendation from respondents was to refrain from calling these studies “cure” research, as this creates artificial expectations and may lead to people consenting without clear understanding. The role of the trial should be explained as being part of the overall development of research on HIV and potential treatment and cures. The actual naming of the trial and its impact on the virus and the participants’ potential well-being will differ from trial to trial, but the naming of the research as the testing of a “cure” has to be avoided until a much higher level of scientific confidence exists.

I don’t think you could tell anybody you’re going to give them an HIV cure. You’re going to say what you’re looking at is trying to reduce the amount of HIV in their body; and that you’re working towards a cure. You certainly can’t put people on the study and tell them that you’re going to cure them; unless you know for sure that you’re going to cure them. (F = HIV researcher)

People are going to have to understand what you mean by cure. How does that define my life? If I have to take the drugs plus this other drug, how do I do it? When do I know about it? When will it be determined that I’m cured? What do I have to do afterwards? (1 = HIV activist)

The inherent risks involved in participation must be understood and highlighted in the information provided. This includes both the risk that the cure may fail and, even if it appears to work initially, that the risk of relapse exists.

Through a good informed consent process…they would acknowledge that this was a risk and that they were willing, they went in with their eyes wide open. (12 = academic)

Present to the patient the fact that there’ve been “X” number of people that have been treated and 1, 2 or 9 patients…have actually experienced this problem. The informed consent form will change so patients will know that there's a good chance of relapse. (6 = HIV researcher)

### Desperation and its impact on risk assessment

Among the respondents drawn from a patient, community or activist base, the desperation for a cure was a consistent theme. Even amongst those who had controlled the virus with HAART, there is a desire to be cured and to end dependence on medication. Desperation for a cure could influence consent processes considerably.

So the ethical concern is will the patients really understand that; and there's always a risk that the patients might be so desperate and be willing to try anything ... even if it’s not in their best interest. (I = social scientist)

I think for any person if you’re going through something as life threatening as HIV there’s always that hope of “I would wake up one day and it’s gone.” So the mere fact that this might be a cure - that in itself is hope and that “I could be cured, I could go back to having a normal life.” (B = academic)

Other respondents, mainly the laboratory researchers, those working on HIV treatment trials and some of the treating physicians, felt that potential respondents would take a considered decision. These respondents felt especially that those who had controlled the virus using HAART and were consistently adherent may not be willing to risk their current diagnostic status and protection to participate in a trial. Although, even in this group, there was an acknowledgement of the desire to be cured.

People will assess their risks and assess what they want to do. It’s going to have to be communicated very well though. (1 = HIV activist)

I suppose there might be a group of patients who would say, well, you know “I'm living happily on my ARVs, you know, why rock the boat?” (B = academic)

### Options for improving information sharing

A number of ideas on methods of improving information sharing and encouraging understanding were shared. There was general agreement for the need to have a strong community education programme in addition to individual consent. Options discussed included the use of pictures and diagrams, or educational videos to aid problems of literacy and to offer a combined verbal and visual display.

You would want your two-page summary, but at the same time it probably isn’t just giving out a brochure. It would be things like having interactive seminars; like what they’ve had in terms of producing a video …and importantly checking understanding, because that’s often lacking in the consent process, …seeing how they understand the risk rather than anything else. So yes, you will probably spend more time on it than you would on any normal study. (D = REC member)

I think more use should be made possibly of pictures rather than words. (B = academic)

Videos would be another way of conveying information. You know, people – I suppose it might be, even more so in the current electronic era; that people's skills with the written word are not that great. And they're more used to pictures, videos, and interactive programmes. (B = academic)

Respondents also suggested that behavioural scientists should make a stronger input into trials so research could be more easily understandable.

The minute it got medicalised we jumped on and then we learnt very quickly that actually doing the medical without the behavioural was folly anyway. (3 = HIV researcher)

Some respondents from a community advisory board suggested training people from the community to act as counsellors and consultants that potential participants could go and talk to. These community informants would be fully informed about the study, and could talk to potential study participants in terms that make sense to them and reflect community issues. These discussions could also happen in a less stressful environment than a research setting.

But people from the community; people whom they trust…like a social worker…I think – because I mean she’s – you can, anybody can explain it to her in English; and she will be able to communicate that to people. (M = religious leader)

It would also be useful to conduct a separate, probably qualitative study, before going into the field to find out what different people need to know, what current beliefs exist about HIV and cure and how these may affect consent, and the capacity of community members to participate in the study if complicated follow-up demands are made.

Then you have a community discussion and really see what people’s thoughts are. If I were you, I would also do a survey with the community; what do you think about this research? (N = REC member)

It is also important to improve assessment of how well participants understood the material. According to respondents, this assessment should also include a component for assessing expectations, especially given the emotions around and desperation for a cure. This would go beyond the usual test of understanding of the consent form that is currently conducted in some HIV studies.

The idea of some sort of questionnaire; be it even a visual one, to assess patients’ expectations if they were to be enrolled into a trial; and to validate any score that might be used as a criterion for entry. (B = academic)

Psychologically I guess you can do some screening, … what the patient’s motivations are; and see if they really understand the risks... And to prepare them for an adverse outcome. (I = social scientist)

A further option raised was to look at consent as a counselling process, where the potential participants are taken through the information in a supportive manner with the research team acting as facilitators to work with the potential participant to make an informed decision.

This kind of informed consent is just not a process of informing and educating the person. It’s about ...it’s actually taking the person through a counselling process. You really need to be taken through the issues like why is it important for you to be part of this research. (1 = HIV activist)

### Consent over a longer time period

Respondents spoke about the implications of having to obtain consent for the extended period of the study, which may be up to 10 or 15 years, to assess if the intervention is a sterilizing or functional cure. Maintaining this relationship over a long trial does have implications. If conditions change years down the trial or the participant realises implications of consent, this has to be taken into account.

You are a participant; you are told things; you haven't done informed consent in the last five minutes, you've done informed consent back at some point a few years ago. You are now three years down in your relationship with this person and you get told stuff. (C = social scientist)

Information provision on HIV, the treatment and the study trial also has to continue over the long-term. As new information emerges about the treatment or about HIV, this has to be communicated to participants, especially if it has to do with their health or risk of a negative outcome.

### Willingness to participate based on trust

Due to the trust participants may place in doctors and other health professionals and in the scientific process, they may be willing to place their trust in the trial without really appreciating the consequences. This trust must not be abused, so all communication must be honest and accurate. Consent in this context does imply a greater dependence on altruism from participants in making their decisions about whether to participate. As stated earlier, a cure is not guaranteed and many early trials are likely to fail. This must be communicated, so that participants understand that their participation is unlikely to bring them an early benefit. Psychological support should also be provided willingly and upfront.

They have trust in the system… and I think we need to be very careful not to abuse that trust. (W = private health funder)

My experience and my work with people is a question of trust …That trust is sort of …on a personal level …they will really open up and they will be willing also to share their experiences if they feel that they can trust. (M= religious leader)

This trust has to be maintained throughout long studies, potentially spanning many years. Therefore, retraining and reinvesting in the relationship will also have to occur regularly.

The challenge with informed consent is that it’s an ongoing process you know so there has always got to be that rapport so they can keep asking questions. (T = HIV researcher)

## Discussion

The ambivalence around HIV exceptionalism with respect to HIV cure research is an important finding and a point of departure in exploring the themes that emerged from this empirical study. Although obtaining consent to conduct HIV cure research will be informed by research ethics guidelines and GCP making it no different from other types of research, most respondents indicated that some aspects of the consent process for HIV cure research are different and will therefore require particular emphasis and consideration. To begin with, consent processes for HIV cure research are different from consent processes for HIV prevention research and HIV treatment research. These differences arise due to the content of information that needs to be shared with potential participants, trial design, interventions, number of early phase research studies available and follow-up. Scientific complexity in cure research generally exceeds the scientific design of prevention and treatment trials [13]. The concept of eliminating the viral reservoir, for example, is unique to cure research. While strict adherence to antiretroviral treatment is stressed in treatment trials, some cure trials may require treatment interruption. The dissonance between what is preached in clinical care and treatment trials and the messaging in cure trials creates an ethical challenge [1]. Furthermore, a homogenous consent process cannot be employed in HIV cure research due to the heterogeneity inherent in HIV eradication strategies. Consequently, consent processes in HIV cure research would differ in accordance with the particular cure strategy employed. Some studies would require analytic treatment interruption requiring detailed explanations of why and how this will occur, others may employ a “shock and kill” strategy with potentially toxic drugs aimed at eliminating reservoirs [13]. Use of a therapeutic vaccine aimed at effecting a durable cure is additionally challenging as patients and potential research participants are more familiar with preventive vaccines than therapeutic vaccines. Gene therapy and bone marrow transplants with HIV resistant donor cells are far more complex scientifically and will require more effort at explanation and translation. This is not a simple matter of HIV exceptionalism. Instead, the requirement for a more robust consent process is directly linked to the nature of the science underlying HIV eradication strategies.

It is therefore not surprising that respondents spoke of the need to go beyond minimal ethical requirements in the case of HIV cure research to address some of the real impacts of HIV at an individual, psychological and community level. Such ethical approaches to HIV cure research need to address these particular concerns at the outset when trials are being designed. At an individual level, PLHIV have experienced stigma, poor health and have lost family members and friends. While current HIV treatments result in longevity, there are costs in terms of side effects and adherence which, in and of itself, are constant reminders of illness. Hence, many infected people are desperate to be cured. At the community level, there have been huge impacts including death and mourning, economic breakdown, orphan-hood, community division and the constant fear of possibly contracting the disease. So, communities as broad systems, both geographical and communities defined by personal characteristics such as men who have sex with men, sex workers or young women, share a high level of vulnerability. This vulnerability means that obtaining individual consent from participants in a trial could easily occur by a quiet manipulation of the process, offering a cure for HIV, creating expectation, taking advantage of the trust in and status of health professionals, especially doctors, and offering incentives of both money and additional treatment. Any perceived benefits of participation play a crucial role in decision-making around enrolment, particularly where the perceived benefits would include the possibility of being cured of HIV [37]. There are many emotions attached to a diagnosis of HIV and rational considerations, such as the potential for risk due to side effects or the development of resistance due to treatment interruption, may not weigh in as heavily. This desperation may lead participants to take excessive risks [38]. Trial medications may have direct risks in terms of adverse effects. Some of these will be anticipated from earlier phase trials and can be explained, but there is the potential for additional unanticipated adverse effects and unknown allergic reactions that may arise. With more radical treatments such as radiation, gene therapy or shock and kill approaches where HIV is lured out of reservoirs and then killed, the risks may be even more difficult to anticipate. Additional risks may occur if the person is on HAART and has to interrupt treatment, or if the commencement of treatment is delayed. Participation may increase rebounding of viral load, and resistance to the treatment used prior to enrolment. There may also be implications for concomitant treatments used for co-morbid illnesses and opportunistic infections, such as tuberculosis and other sexually transmitted infections. Hence the need expressed by respondents for clear communication of risks and benefits.

The potential for manipulation of trial participants based on suboptimal risk perception requires a level of protection and additional oversight from RECs and from researchers conducting studies. Others have argued, based on decision-science, that participants should be allowed to accept research-related risks in keeping with their personal risk profiles for other aspects of their lives [39]. While this may be acceptable in highly educated, empowered communities, it may not work in educationally disempowered vulnerable communities in low resource settings. The intersection of individual good and community good also plays itself out in terms of a risk-benefit calculation. Dan Wikler argues that federal research ethics guidelines in the United States often refer to individual risk versus community benefit. Yet others calculate risk relative to individual benefit. The global health value of finding a cure is so substantial that additional individual risk would almost be justifiable provided participation is voluntary and fully informed [40]. This view is expressed in South African research ethics guidelines where the analysis of risks and benefits needs to consider participants but community and societal interests as well [41].

Respondents in this study argued strongly that HIV research studies should not offer or claim a cure. This sentiment has been expressed by others. George Annas refers to “cure research” as an oxymoron [42]. The level of science currently makes what people envisage as a cure- a permanent eradication of the existing virus – unlikely [43]. Sylla et al. similarly found in their study that respondents “conceived of a cure as eradicating”. Many participants “did not regard functional cure as an improvement over controlling HIV through ART” [22]. Consequently, “cure” research may be best presented as research that is part of a process towards finding a cure. This does present a real challenge in the field as the difference in position is subtle and study staff involved in recruitment can easily slip into the narrative of cure. Others have suggested that deliberate use of specific terms such as “experiment” be adopted to guard against curative misconception [44]. This is the language that RECs need to be attentive to in reviewing consent documents for so-called HIV “cure” studies. The first HIV cure study conducted in the Western Cape did not use the word cure in its consent information documents (personal communication). This is consistent with the views expressed by respondents who urged caution in using the word cure in experimental clinical trials.

As argued above, the best defence against curative misconception is the provision of sufficiently clear and easily understandable information so that potential respondents know what they are committing to. This is more complex than the statement implies. A number of ideas are presented by respondents for providing this information including educational videos, cartoons and pamphlets. This implies that RECs need to ensure that various consent tools are submitted to enhance understanding during the consent process in proposed HIV cure trials.

This study has highlighted a unique dimension of consent in the context of HIV cure research. Previous research has highlighted how participants conceptualise risks in HIV cure research as having biological, psychological and social components [26]. HIV cure research would offer a biological cure only. Healing, as a holistic construct, embodies biological, psychological and social components in restoring patients to health. There is therefore a need to reframe biological cure as a component of healing, and additional attention must be paid to the psychological and social elements to complete the process of healing on the path to HIV elimination. This has implications for how the REC reviews both the protocol and consent process for HIV cure research. Researchers ought to be required to submit a plan to the REC outlining how psychological and social harm will be mitigated in the context of high risk HIV eradication trials involving vulnerable populations desperate for a cure. In addition to science translation to facilitate understanding of consent information, respondents referred to assessment of psychological readiness to consent to participate in cure research. This would ensure that desperation for a cure did not result in underestimating the risks involved. Furthermore, in the event of failure of an intervention to lead to cure, psychological support and counselling would be necessary. These concerns are echoed in the work of Sylla et al. where participants were “distrustful about viral rebound potential” and the “accompanying psychological distress” [22]. This could occur a few years post trial and unlike other studies, long-term consent processes and support services would be necessary. While ideal, it may not always be practical to have a psychologist involved in a clinical trial. In this case other counsellors, adequately trained to address the psychological well-being of trial participants and to provide counselling in the event of negative trial results for cure interventions. RECs would need to request evidence of the availability of such counsellors both during a cure trial and after the study.

With long-term research studies, participant education may need to occur on a phased basis. This would require the development of decision-making tools so a decision can be broken down into components such as safety of the intervention, ancillary care that would be provided, benefits to the person, benefits to society and community, nature of treatment, and how the intervention works. By presenting all of these components separately it may be easier for the potential participants to develop a clearer picture of what the trial is about.

Another core consideration in the type of research anticipated here is that the consent process has to be embedded in a long-term relationship between the research team and participants. This has implications for consent information. It has to be clear at the outset that the potential participant is being recruited for a trial that will include ongoing monitoring that may last 10 years or more. This is not unusual as many cohort studies extend for much longer, but in the case of HIV cure research there are potentially risky drugs involved, coupled with high levels of expectation. RECs would have to request follow-up consent templates in addition to the enrolment consent form. One consideration here would be to have the participants sign an annual re-consent to their participation in the study. This would be in line with the study having to request renewal of REC approval annually. In the event that some “cure” trials are regarded as “high risk”, more frequent REC monitoring (such as 6 monthly progress reports) may be indicated.

This study sought to elicit a broad range of perspectives from multiple HIV stakeholders in three major regions of the country. Consequently, the diverse views expressed by participants have made a meaningful contribution to this emerging field of study in South Africa.

An important limitation however is that HIV cure research has not started in earnest in South Africa and hence our discussions revolved around hypothetical trials. Perceptions may change when the prospect of cure becomes a reality. Furthermore, only one interview was conducted per participant due to sample size and time constraints. The potential for further longitudinal studies exists. While the absence of guidelines on the ethics of HIV cure research is a challenge, empirical research from this study has the potential to inform future guideline development.

## Conclusion

Against the backdrop of HIV prevention and treatment trials in South Africa over the past three decades, HIV cure research is generally perceived to require unique consent approaches. While significant efforts would need to be invested in science translation to maximise understanding of trial procedures, risks and the need for long-term follow-up, the psychological dimension of cure should not be underestimated. Beyond an understanding of cure science, the emotional impact of HIV resulting in desperation, hope and expectation, advances the discourse from cure to healing. Consequently, the consent process for cure research would need to be significantly enhanced to include psychological support and counselling. Ethics review requirements for consent in HIV cure research will need to examine the language in consent documents more closely to ensure that eradication messaging is not misleading. Consent requirements may need to be augmented to accommodate additional measures such as educational tools to enhance participant understanding and to ensure psychological well-being during recruitment, enrolment and long-term follow-up. Debriefing of trial participants would be necessary at regular intervals. Re-consent in long-term studies, more frequent progress reports and post-approval monitoring of HIV eradication studies are strongly recommended. Finally, the development of a specific guidance document for HIV cure research would assist and expedite the REC review process to enable complex scientific eradication research to proceed to the mutual benefit of researchers, participants, communities and societies.

## Abbreviations

FGDs: Focus Group Discussions
HAART: Highly Active Antiretroviral Treatment
HIV: Human Immunodeficiency Virus
IDI: In Depth Interviews
NHREC: National Health Research Ethics Council
NIH: National Institutes of Health
PLHIV: People Living With HIV
REC: Research Ethics Committee
